# Correction: Impact of emotional labour on taking charge to predict employee’s creative and task performance: The moderation of performance-based pay from the lens of self-determination theory

**DOI:** 10.1371/journal.pone.0315774

**Published:** 2024-12-11

**Authors:** The PLOS ONE Staff

## Notice of republication

This article was republished on November 25, 2024, to correct an error with Fig 1. Please view Fig 1 here. The publisher apologizes for the errors. Please download this article again to view the correct version. The originally published, uncorrected article and the republished, corrected articles are provided here for reference.

## Supporting information

S1 File Originally published, uncorrected article. (PDF)

S2 File Republished, corrected article. (PDF)
