# Supplementary material for: Correction: Impact of emotional labour on taking charge to predict employee’s creative and task performance: The moderation of performance-based pay from the lens of self-determination theory
Source: PLoS One. 2024 Dec 11;19(12):e0315774. doi: 10.1371/journal.pone.0315774 (PMC11633983; doi:10.1371/journal.pone.0315774)
Supplement: S2 File — (PDF) [file pone.0315774.s002.pdf]

PLOS ONE

RESEARCH ARTICLE

Impact of emotional labour on taking charge to predict employee's creative and task performance: The moderation of performance-based pay from the lens of self-determination theory

Nilesh Kumar[1,2]*, **Zhiqiang Liu**[2], **Carol Flinchbaugh**[3], **Md. Yahin Hossain**[2,4], **Md. Nahin Hossain**[5]

1 School of Business Administration, Zhejiang Gongshang University, Hangzhou, China, **2** School of Management, Huazhong University of Science and Technology (HUST), Wuhan, China, **3** Department of Management, New Mexico State University, Las Cruces, NM, United States of America, **4** Department of Business Studies, University of Information Technology and Sciences, Dhaka, Bangladesh, **5** Army Institute of Business Administration Savar, Savar, Bangladesh

* nileshk@live.com

Abstract

The importance of emotional labouring and performance of frontline service employees, who in their boundary-spanning positions significantly affect service-rendering organisations' efficiency by their direct communications with customers, continues to increase. However, it is still important to ascertain an efficient understanding of the comprehensive process including behavioural mechanism and a common perception of the rewards' impacts on motivation and creativity. Therefore, guided by self-determination theory, this study examined the mechanism and boundary conditions between emotional labour and job performance (creative and task)–specifically, taking charge has been considered as a mediator and performance-based pay as a moderator in between relationships. The authors selected a time-lagged cross-sectional design to investigate interrelations amongst study variables at two different time points and surveyed 417 team members and 186 team leaders in Pakistan's commercial banks. Findings were consistent with the assumed conceptual framework. For instance, deep-acting affected taking charge positively, surface-acting demonstrated a positive link with task performance and taking charge partially mediated the relationships between deep-acting and performances under boundary conditions of low performance-based pay. By summing up, the study adds to the literature and recommends managerial implications with a more affluent view of nomothetic linkage among frontline employees' emotional labor, HR practices, and the service sector.

OPEN ACCESS

Citation: Kumar N, Liu Z, Flinchbaugh C, Hossain M.Y, Hossain M.N (2022) Impact of emotional labour on taking charge to predict employee's creative and task performance: The moderation of performance-based pay from the lens of self-determination theory. PLoS ONE 17(10): e0269196. https://doi.org/10.1371/journal.pone.0269196

Editor: Andrea Fronzetti Colladon, Universita degli Studi di Perugia, ITALY

Received: October 5, 2021

Accepted: May 17, 2022

Published: October 6, 2022

Copyright: © 2022 Kumar et al. This is an open access article distributed under the terms of the Creative Commons Attribution License, which permits unrestricted use, distribution, and reproduction in any medium, provided the original author and source are credited.

Data Availability Statement: The datasets used during the current study cannot be shared due to respondents' privacy concerns. As such, participants were assured that data will only be used for research purpose and will not be shared to third party for any reason. Therefore, data cannot be publicly shared. However, those who are interested in data sets can request specially to Dr. Nilesh Kumar or Ethics Committee of Zhejiang

Gongshang University on 2021160@mail.zjgsu.edu.cn.

Funding: The author(s) received no specific funding for this work.

Competing interests: The authors have declared that no competing interests exist.

1. Introduction

In an increasingly evolving and uncertain business environment, service organizations are engaging themselves in updating their competencies for improved performance and creativity [1, 2]. For labor-intensive and interactive-services, individuals as authentic service providers are example of improvement, change, and innovation [3]. Employee's service delivery process and creative insights ensure that their organizations can create a superior customer experience, adapt ever-changing business environment, and identify innovative opportunities for improving performance [2, 4, 5]. This implication of an employee's effective service delivery and creativity commands us to ascertain an efficient understanding of the comprehensive processes including its predecessors, outcomes, and controlling factors.

Given that effective customer service and the origination of constructive ideas regarding service procedures and practices are mainly determined by the workplace environment [5–7], it is, therefore, practical to approach employee service delivery and creativity with context-specific perceptions. Service employees are usually thought of as having to comply with behavioural rules set by their organisations and are mainly bound to carry out emotional labour to convey expected emotions [8–12]. Hence, emotional labour as an obligatory job-related demand in the service sector might be a context-specific predecessor of employee creativity and task performance [13–15].

Emotional labour refers to individuals' self-regulation of internal feelings and external expressions, or the process of planning, controlling, and displaying desired emotions while dealing with customers according to the organisational display rules [9, 11, 16]. Earlier studies asserted that emotional labour is an effortful practice that drains inadequate resources and has adverse consequences [11]. However, later research has demonstrated that two components of emotional labour (e.g., surface acting and deep acting) have a diverse impact on personal and job-related outcomes i.e., [17–21]. In a similar way, some researchers have investigated the relationship between emotional labour, employee creativity, and service performance i.e., [7, 13–15, 22–24]. Although it has been proposed that both components of emotional labour are differentially related with employee creativity and service performance, little is known about any behavioural mechanisms between them. For example, Geng et al. [13] examined the psychological state (e.g., challenged stress) as the mechanism between deep acting and employee creativity. Building on emotion-regulation theory, Shin, Hur and Oh [15] investigated affective commitment and emotional exhaustion as mechanisms between deep acting and employee creativity. In a similar way, Jaewon et al. [24] indicated work engagement as a mechanism between deep acting and job performance. In studies that followed, all the mechanims were either psychological or self-regulated responses. Therefore, to precisely understand the association between emotional labourers' approaches and their performances (creative and task), it is crucial to study behavioural responses or the mechanism between both. As such, it is reasonable to debate how emotional labourers are apt to bring creative solutions and ideas for their organisations and perform assigned duties [7, 25, 26]. On the basis of past studies e.g., [13, 15, 20, 23, 24, 27], our study, therefore, emphasises proactive behaviour and motivational factors that function to encourage employees and keep their attitudes and feelings attached to their organisation. More specifically, it examines the mediating role of taking charge behaviour in between emotional labour strategies and creative and task performances from the lens of self-determination theory-SDT [28].

Taking charge has been described as both voluntary and creative efforts performed by individual workers with the intent to effect constructive change concerning job completion. This assumes that taking charge is a crucial type of proactive behaviour that maintains organisational survival and individual growth [29–31]. Empirical evidence has shown that individuals

have an inherent need to struggle for consistency [32–34]. As a result, individuals strive to adjust their emotions and keep their attitude consistent. From the perspective of self-determination theory, when people are motivated autonomously, they practice volition or a self-endorsement of their actions. Autonomous motivation promotes a deep acting approach, a taking charge attitude and other proactive behaviours e.g., [30, 31, 35–37]. Therefore, it is expected that deep acting engages in taking charge behaviour to foster creative and task performance, and surface acting doesn't engage in taking charge because of its disposition of controlled motivation. This is because taking charge is a flexible and impulsive type of committed behaviour, which is based on autonomous motivation, not controlled motivation [29]. Nevertheless, to our knowledge, no study has so far considered the relationship between emotional labour and taking charge. Therefore, considering the similar evidence of both emotional labour and taking charge, we build their undiscovered relationship to fill a gap in the literature.

Additionally, some reseachers with an interest in the impacts of taking charge and emotional labour presume that both are beneficial to creative and task performance when there is a high amount of pay and wages e.g., [25, 38]. But others equally posit that pay and financial rewards diminish intrinsic motivation [39, 40] and state that creativity comes from an intrinsic/autonomous motivation [41, 42], not controlled motivation and financial rewards [43, 44]. Even after years of study, this disagreement has not offered consistent guidelines or a common perception of the rewards' impacts on motivation and creativity; therefore, scholars have called for further research to fill this gap [43, 45, 46]. Likewise, scholars have tended to pay closer consideration to defining taking charge behaviour's predecessors and outcomes and have tended to pay less consideration to its sustainability [41, 47, 48]. In this sense, considering a moderation role of performance-based pay will also fill a gap in the literature noted by Baroundi et al. [38] and Grandey and Sayre [25]. Therefore, guided by self-determination theory, we considered performance-based pay as a moderator on our direct and indirect relationships to identify how performance-based pay captures the benefits of emotional labour in driving job performance through taking charge.

In sum, we addressed concerns by testing a theoretical framework (see Fig 1) in the context of the banking sector and a developing country (Pakistan). In demonstrating the relationships

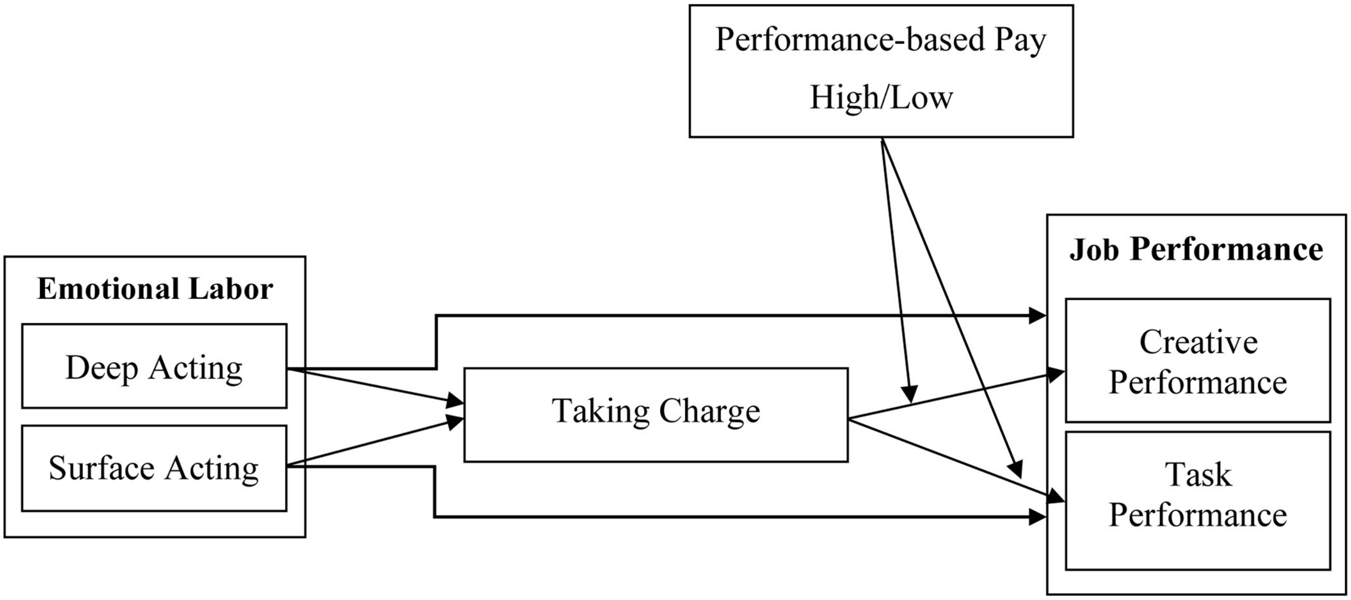

Fig 1. The hypothesized model.

https://doi.org/10.1371/journal.pone.0269196.g001

proposed in a given model and by answering the above questions, our outcomes may advance existing literature on emotional labour, proactive behaviours, compensation and creative performance in several ways. First, we attempted to illuminate different effects of emotional labour on taking charge from a self-determination perspective. Second, we added a new understanding by extending emotional labour outcomes to job performance from a taking charge perspective. Third, we clarified that the level of performance-based pay acted as a moderator that provided valuable knowledge necessary for engaging in creative and task performance through taking charge. Additionally, our study's outcomes and recommendations will not merely be put into action in the banking sector but could also be implemented in other relevant sectors. Governing bodies will make use of this research as it will offer complimentary evidence useful in the formulation of a compensation plan and policies. In addition, it will help the regulatory framework–specifically in labour-management practices–by considering the motivation level of emotional labour, individuals' involvement within the organisation, and individuals' creative approaches under the boundary condition of performance-based pay.

2. Literature review and hypotheses development

2.1 Emotional labour, taking charge and job performance (creative and task)

According to self-determination theory, the reason to engage in emotional labour is not simply an issue of quantity of motivation but also an issue of quality of motivation [49]. Self-determination theory differentiates between two categories of intentional behaviour: controlled and autonomous [28, 49–52]. Since emotional labour is predicated on an intentional action [53], this difference could assist us to inquire further as to why service employees adopt certain emotion-regulation strategies. SDT posits that service employees enage in emotional labour because they feel an internal or external pressure to do a task or because they need to adjust their emotions. Additionally, SDT posits that there are distinctive forms of motivation aligned on a continuum, which differentiates autonomous motivation from controlled types of motivation [28, 49, 50]. There is then amotivation at one end of the continuum framework [28, 49, 50]. Amotivated individuals are those who adjust their feelings without their desire and with resignation. They may not adjust their emotions or feelings at all, but if they do, they do not feel that they have control over their emotional displays. Both types of emotional labour are, therefere, regarded as motivation-based strategies utilised by service employees [37]. Emotional labour strategies are categorised into autonomous motivation (i.e., deep acting), which refers to adjusting internal feelings by altering a situation or perceptions of a situation, and a controlled motivation form (i.e., surface acting), which refers to faking emotions where one is not naturally involved or intrinsically motivated, or amplifying an emotional response to carry out a task under external or internal pressure [9, 18, 37, 54].

Building on past studies that disclosed that different characteristics of deep acting and surface acting are likely to generate significantly distinctive effects on service employee outcomes, including engagement, proactiveness, attitude, behaviors, and performances [13, 20, 22–24, 27, 55], we posit that service employee's acting strategies will differentially influence their level of creative and task performance. In other words, it is assumed that deep acting will facilitate employee creativity and that surface acting will hinder it, whereas both will be conducive to task performance. However, it is evident that both service employee's acting strategies and performance types are motivation- and behaviour-oriented constructs. It is not easy to conceive that an employee's motivation and behaviour directly invite subsequent behaviour. This obliges us to recommend comprehensive justifications for these phenomena and to investigate definite mechanism among constructs. Therefore, we seek to ascertain the mediating process

that accounts for the association between emotional labourers' intentional behaviour and performance types–meaning the creative and task performance and HR practices that sustain and moderate the relationship.

Given that emotional labour is an outcome of an individual's level of internalization (introjected and integrated), undertaken to direct subjective states [35] and that their internalization plays a key role in exhibiting creative and task performance [37, 40], emotional labourers' internalization levels seems to function as intervening factors among emotional labour components and performance types (creative and task). Our research, therefore, attempts to explain the mediating role of internalization levels in terms of taking charge behaviour. Taking charge behavior can best be understood as 'employees' voluntary and constructive efforts to effect organisationally functional change with respect to how work is executed within the contexts of their jobs, work units, or organisations' [30, p. 403]. Our paradigm of taking charge is where service employees offer ideas to ascertain a business opportunity or to improve a situation relating to their job tasks and possibilities [56]. The main concern here is that this kind of behaviour improves certain work-related situations or events or permits service employees to proactively shape their careers according to their values and goals [57]. In particular, it aligns with SDT, which posits that individuals have fundamental human needs and set objectives in life in order to satisfy those needs [50]. This theory is exceptionally noticeable for deep actors who like to set their personal career and life goals [35]. Deep acting labourers influenced by autonomous motivation are growth-oriented and are thus apt to engage in taking charge behaviours to attain their maximum potential, whereas surface acting labuorers influenced by controlled motivation don't engage in these behaviours [37, 58, 59].

When a mediating role is examined, our research notes that the two types of employees acting have different effects on taking charge. The faking of required emotional expression and the suppression of true emotions involved in controlled motivation–such as surface acting–requires the demonstration of an emotional façade, which lessens feelings of authenticity [8, 35, 54]. The social interaction model by Coté [60] recommends that this inauthenticity stimulated by surface acting brings about harsh feedback and adverse reactions from customers. However, according to self-determination theory, surface actors are not inherently motivated and satisfied to perform a job they still perform to avoid negative consequences such as a punishment (e.g., a deduction in pay), and to attain positive outcomes such as a reward (e.g., a bonus) [35]. This propels employees towards extrinsic motivation [50]. Thus, they see money or a salary as a motivation to accomplish assigned work [40, 44, 49, 51]. Indeed, it seems that surface acting has disruptive effects, and deep acting appears to produce positive results e.g., [13, 15, 20, 24, 27]. More specifically, deep acting has been positively associated with organisational citizenship behaviour [20], task performance, contextual performance, innovative job performance [22], and frontline employee creativity [13]. And compared to surface acting, deep acting demonstrates less emotional exhaustion and more effective committed behaviour, which produces a high level of creativity [15] and work engagement [24]. Thereby, deep actors' authentic displays of emotions might benefit both the individual and the organization by promoting more positive communication between employees and customers.

Hur et al. [37] provided clear and strong empirical evidence on this phenomenon. They demonstrated that autonomous motivation positively associated to subsequent deep acting and controlled motivation were positively associated with surface acting. Specifically, service employees with autonomous motivation were likely to concentrate their attention and emotional resources on work-related activities that were aligned with their personal identity, values, and interests [49], thereby allowing employees to willingly adjust their emotions to organisationally desired displays. Given that autonomous motivation leads to voluntarily reliable behaviour [61], deep acting should have a significant relationship with taking charge that

could maximise correspondence between one's internal emotions and creative and task performance. Thus, autonomously motivated individuals, termed as deep actors, are more likely to engage in taking charge than surface actors, and are likely to show considerable creative and task performance.

Taking charge is described as both a voluntary and creative efforts performed by individual workers with the intent of effecting constructive change regarding job completion. It is an example of a worker's potential to assist in diverse roles to transform his or her workplace [62]. A key factor of this kind of behaviour is that it is innovative and change-oriented. Moreover, it pushes individuals to be more adaptive [59]. Taking charge is a crucial type of proactive behaviour that maintains organisational survival and promotes individual growth. It has a number of practical uses. Constructing new processes to carry out job duties, changing the approach to job performance to amplify efficiency, or making on-the-spot modifications of substandard practices or procedures are all practical uses of taking charge [30]. Empirical evidence shows that individuals have an inherent desire for consistency [32, 33]. As a result, individuals strive to adjust their emotions and keep their attitude consistent. From the perspective of self-determination theory, when people are motivated autonomously, they practice volition or a self-endorsement of their actions. Autonomous motivation promotes the deep acting approach, a taking-charge attitude, and other proactive behaviours e.g., [30, 35–37]. Therefore, it is expected that deep acting would lead to taking charge behaviour to foster creative and task performance, and that surface acting wouldn't result in taking charge because of its disposition of controlled motivation. Thus, taking charge is a flexible and impulsive type of committed behaviour, which is based on autonomous motivation, not controlled motivation [29]. In addition, employees' taking charge facilitates better performance evaluation, affective commitment, and job satisfaction [47, 48] as well as improves leadership potential, builds social networks, and promotes creativity [57].

The demonstration of creative performance generally involves substantial interest and desire [63], and service employees who are emotionally committed to their organisation are inclined to attempt to be good in ways that are compatible with their organisation's goals [64]. Given that modern organisations in the service industry are likely to emphasise innovation for long-term sustainability and growth, employees who bond strongly with an organisation and align themselves with their organisation's aims and objectives [65] are mostly prepared to contribute towards their organization's success and growth by creative behaviours. In particular, Kumar et al. [41] posited that since creative performance demonstrates a form of engagement that generates constructive ideas for developing business, taking initiatives, challenging the status quo of an organisation, engaging in voluntary activities, presenting it in systems, products, and services is a critical indicator of an employee's taking charge within an organisation. They quantitatively exhibited that motivated employees demonstrated a high level of creativity and task performance by engaging in taking charge behaviour. In terms of task performance, it has been found that surface acting has disruptive effects, and deep acting appears to produce positive results e.g., [13, 15, 20, 24, 27]. Although past studies have examined the negative side of surface acting with creative and task performance see [13, 15, 24, 27, 37], we found support from forms of SDT-controlled motivation, namely surface acting, which refers to faking emotions or amplifying an emotional response to carry out an assigned task to avoid negative consequences [9, 18, 37, 54]. Moreover, according to Judge et al. [66] and Pugh, Groth, and Henning-Thurau [67], surface acting is not always detrimental, which indicates that it should demonstrate a positive relationship with task performance. Therefore, taken together with observed evidence, we assume the following propositions:

Hypothesis 1: *Emotional labor affects taking charge, but the effect is different; specifically,* ***(a)*** *deep acting affects taking charge positively, and* ***(b)*** *surface acting affects taking charge negatively.*

Hypothesis 2: *Emotional labor affects job performance, but the effect is different; specifically, deep acting has a positive effect on **(a)** creative performance and **(b)** task performance, and surface acting affects **(c)** task performance positively but **(d)** creative performance negatively.*

Hypothesis 3: *Deep acting has an indirect positive relationship with the **(a)** employee's creative performance and **(b)** task performance via taking charge, but **(c)** the effect on creative performance is stronger.*

2.2 Performance-based pay as a boundary condition

Performance-based pay is a compensation system that depends on a pre-determined level of performance. It includes financial and non-financial rewards, e.g., pay, promotion, and other types of compensation [68]. Rewards drive employees' morale, and most workplace incentives are driven by one's performance appraisal [69]. These incentives are known as performance-based pay and are believed to boost individual productivity and work quality [70].

Indeed, individuals manage their feelings and display emotions for pay [11, 39]–that is why pay is considered as a management practice, implemented to control employees' interests while positively influencing performance [71]. It is effective in manipulating work behaviours such as risk-taking (related to taking charge). Taking charge has a positive impact on an employee's performance [41] and career satisfaction, which ultimately benefits an organisation and enhances overall performance [72, 73].

Past studies have discovered that pay, emotional intelligence, and job autonomy can increase workers' taking charge behaviours [38, 47, 74]. Other researchers have examined performance-based pay's role in fostering workers' taking charge behaviours. Previous studies in the fields of compensation e.g. [75] and economics e.g. [76, 77] have revealed that risk aversion impacts employees' attitudes towards performance-based pay. It is an essential aspect in explaining the effects of financial incentives on employees' creativity and willingness to take risks (related to taking charge) [78]. Another study posited that employees are more likely to be expected to engage in taking charge when they get an increase in pay [38].

However, according to Deci and Ryan [40, 79], rewards have a tendency to be experienced as controlling, which can induce or pressure an employee to perform differently from how they would act freely. Controlled motivation can promote improved performance, but creativity comes from autonomous motivation, self-consciousness, and self-actualization [41, 42], rather than from controlled motivation or financial rewards [40, 44]. Consequently, when individuals feel controlled by extrinsic rewards, they experience pressure to think, behave, or feel in specific ways [51]. Rewards contingent upon performance increase an employee's pressure [80] and stress [81], which can lead to lower creativity. Furthermore, performance-based pay promotes repetition of work that has been done in the past, instead of innovation and exploration of new, untested approaches [82–84]. In a similar vein, Fischer et al. [43] also found no statistical association between transactional rewards and intrinsic motivation. Therefore, taken together with observed evidence, we assume the following propositions:

Hypothesis 4a: *The relationship between taking charge and creative performance is weaker for individuals with high performance-based pay.*

Hypothesis 4b: *The relationship between taking charge and task performance is stronger for individuals with high performance-based pay.*

Hypothesis 4c: *Performance-based pay moderates the indirect relationship between deep acting and creative performance via taking charge, such that the relationship is amplified when performance-based pay is low.*

***Hypothesis 4d**: Performance-based pay moderates the indirect relationship between deep acting and task performance via taking charge, such that the relationship is amplified when performance-based pay is high.*

3. Research methodology and analysis

3.1 Sample and procedures

The study is explanatory research; thus, we selected cross-sectional design to investigate interrelations amongst study variables at two different time points and surveyed 417 team members and 186 immediate team leaders in the banking sector in Pakistan. Employees' job descriptions were to attract new customers for various services offered by the bank, sell benca-products, bring targeted deposits, engage in PR drives, and deal with complaints. We distributed questionnaire survey, using convenient sampling technique, as data collection tool and inimized potential of common method biasness by collecting data from two different sources. In the first phase, we collected responses from team members on emotional labour (surface and deep acting), taking charge, and performance-based pay. In the second phase, after 1 month, we asked team leaders to rate for their team members' individual-level creative and task performance.

To attain the study's research objectives through statistical analysis, we used reliability and validity of data and structural equation modeling to evaluate the assumed hypotheses by using SmartPLS and SPSS Process-Macro. At first, we employed "PLS Algorithm" to identify data-analysis of measurement model (outer framework), e.g., reliability of items, convergent validity, discriminant validity, and model fitness. Later, we used structural equations modeling (SEM) via Process v3.4 (IBM SPSS add-on) by Hayes [85–87]. Moreover, we received 417 responses. Further details are demonstrated in Table 1.

3.2 Measures

We used already developed measurement scales and ensured their reliability and validity in the current study. In addition, we used seventh point Likert scale to get responses for all measurement scales; options ranged from 1 = Strongly Disagree to 7 = Strongly Agree.

Table 1. Demographics composition of respondents or participants.

Variables	Response Category	Frequency	Percentage
Gender	Female	87	20.9%
	Male	330	79.1%
Age	"20–25 years"	81	19.4%
	"26–35 years"	240	57.6%
	"36–45 years"	48	11.5%
	"46-Above"	48	11.5%
Education	"Diploma"	18	4.3%
	"Bachelors"	219	52.5%
	"Masters"	180	43.2%
	"Ph.D."	0	N/A
Tenure	"Less than one year"	48	11.5%
	"1–3 years"	165	39.6%
	"3–5 years"	78	18.7%
	"5–8 years"	54	12.9%
	"Above 8"	72	17.3%

https://doi.org/10.1371/journal.pone.0269196.t001

Emotional labor. We used six items from Emotional Labor scale developed by [54] to measure surface acting (3 items; e.g., "I pretend to have emotions that I do not really have") and deep acting (3 items; e.g., "I make an effort to actually feel the emotions that I need to display to others"). Cronbach's alpha coefficients for this research are; .75 for surface acting and .70 for deep acting.

Taking charge. We used [30] ten-item scale to measure taking charge. Taking charge as a construct represents proactive, change, or challenge-oriented forms of citizenship [88, 89]. Since this variable was self-reported thus, we modified it according to study's objectives. The sample item is, "I try to institute new work methods that are more effective for the company." Cronbach's alpha coefficient for TC in this research is .77.

Performance-based pay. We used Pay For Performance scale developed by [90] with three-item and modified reverse coded items. The sample item is "My individual performance actually has a great impact on any incentive pay award," Cronbach's alpha coefficient for PBP in this study is .71.

Creative performance. We used Innovative Job Performance scale developed by [91] to measure creative performance. As earlier clarified, creative and task performance of individuals in the team were rated by their team leaders to avoid possible common method bias. Furthermore, the scale was modified as per flexibility of the survey. These items include, does this worker perform the following work activities? And used following clauses for rating purpose. For instance, "Creating new ideas for improvement"; "transforming innovative ideas into useful applications." These items assess that employee performs creatively. Cronbach's alpha coefficient for CP in this study is .86.

Task performance. We used three items drawn from [92] to measure task performance. The sample item is "This employee adequately completes assigned duties". These items assess tasks which employees are expected to perform regularly. Cronbach's alpha coefficient for TP in this study is .78.

Control variable. Empirical research has found links between both demographic variables. Thus, we added gender, age, education, and tenure as control variables. We measured education on a four-point scale (1 = "Diploma," 2 = "Bachelors," 3 = "Masters," 4 = "Ph.D."), tenure level on a five-point scale (1 = "Less than one year," 2 = "1–3 years", 3 = "3–5 years", 4 = "5–8 years", 5 = "Above 8"), age level on a four-point scale (1 = "20–25 years", 2 = "26–35 years", 3 = "36–45 years" 4 = "46-Above") and gender as a dichotomous dummy variable (1 = female and 2 = male).

3.3 Ethical consideration

Participants were informed prior to the survey regarding the purpose of research and were given assurance for the confidentiality of data. All participants responded voluntarily and those who completed form, their completion was considered as consent. Such that, the study was conducted in line with Helsinki Declaration principles. We used standard procedures and measurement instruments and sought approval from academic development and ethics committee of Zhejiang Gongshang University (Reference No. 202108/IRB/54).

3.4 Data analysis

Data were analyzed in two stages; at first, we identified basic examination of data of measurement model (e.g., reliability of items, their convergent validity, discriminant validity, and also model fitness). Secondly, after suitable results, we assessed structural equation modeling followed by model no. 04 for simple mediating effect and model no. 14 for the mediated-moderation effect through process-macro. In the whole process, individual-level data were used to test the proposed relationships.

Table 2. Construct reliability and validity.

Main Variables	Cronbach's Alpha	Composite Reliability	Average Variance Extracted (AVE)
Surface Acting	.75	0.720	.575
Deep Acting	.70	.736	.607
Taking Charge	.77	.776	.637
Performance-based Pay	.71	.753	.548
Creative Performance	.86	.779	.671
Task Performance	.78	.864	.706

https://doi.org/10.1371/journal.pone.0269196.t002

3.5 Construct reliability and validity

In this study, the consistency of participants' responses to the survey questionnaire were checked by evaluating the reliability of measurement items. Our study, therefore, performed a reliability analysis of 6 constructs by using Cronbach's alpha (α) and it found that Cronbach's value is $\alpha > 0.7$ for all constructs. We also assessed convergent validity. According to Hair Jr. et al. [93], the ideal standardized average variance extracted (AVE) should be greater than 0.5, and reliability must be greater than 0.7 to demonstrate sufficient convergent validity. Therefore, based on outcomes, values of all variables are higher than cut-off criteria and threshold values, which show quite good internal consistency. Essentially, measures were satisfactorily found reliable to proceed with further analysis. The following Table 2 illustrates outcomes of reliability and validity.

3.6 Model fitness statistics

We performed confirmatory factor analysis (CFA) on six constructs and tested the fit of a six-factor model; surface acting, deep acting, taking charge, performance-based pay, creative performance, and task performance. Table 3 shows that hypothesized six-factor confirmed acceptable fit ($X^2(120) = 170.19$, $p < .005$; RMSEA = 0.06, CFI = .94). Additionally, all the factor loadings were significant, sustaining convergent validity in this research. Meanwhile, to check the discriminant validity of our calculation, the model fit of hypothesized six-factor model was weighed against the sequence of alternative models. As presented in Table 3, six-factor model fits the data finest, recommending support for distinctiveness of the variables.

Table 3. Model fitness statistics.

Model	X^2	*Df*	X^2/Df	*SRMR*	*RMSEA*	*CFI*
Six-factor model (Default model)	170.19	120	1.42	.06	.06	.94
Five-factor model [a]	268.80	125	2.15	.11	.09	.83
Four-factor model [b]	326.57	129	2.53	.11	.11	.76
Three-factor model [c]	434.97	132	3.30	.13	.13	.63
Two-factor model [d]	458.99	134	3.43	.14	.13	.60
One-factor model [e]	584.05	135	4.33	.16	.16	.45

Note: CFI = Comparative fit index; RMSEA = Root mean square error of approximation; SRMR = Standard root mean residual

[a] Performance-based pay and Surface acting were loaded on one factor

[b] Surface acting, Deep acting and Performance-based pay were loaded on one factor

[c] Surface acting, Performance-based pay, Deep acting and Taking charge were loaded on one factor

[d] Surface acting, Performance-based pay, Deep acting, taking charge and Creative performance were loaded on one factor

[e] All variables were loaded on one factor.

https://doi.org/10.1371/journal.pone.0269196.t003

Table 4. Means, standard deviations and correlations.

Variables	Mean	SD	1	2	3	4	5	6	7	8	9	10
1. Gender [a]	1.790	.408	-									
2. Age	2.150	.867	.295**	-								
3. Qualification	2.390	.571	-.85	-.236**	-							
4. Tenure	2.850	1.29	.270**	.668**	-.284**	-						
5. Surface acting (EL) [b]	4.245	1.59	.176*	.205*	.086	.176**	**(.75)**					
6. Deep acting (EL) [b]	4.374	1.04	.114	.140	-.022	.066	.200*	**(.70)**				
7. Taking charge [b]	5.175	1.15	.006	.133	.149	.104	.007	.468**	**(.77)**			
8. Performance-based pay [b]	4.925	1.33	.064	.181*	-.107	.090	.074	.344**	.171*	**(.71)**		
9. Creative performance [c]	5.498	.117	.083	.068	.066	.073	.154	.496**	.425**	.306**	**(.86)**	
10. Task performance [c]	5.413	1.03	-.018	.021	.075	-.026	.178*	.405**	.393**	.333**	.634**	**(.78)**

Note: *ns* = 417 individuals from 186 Teams.

[a] Female = 1, Male = 2

[b] Rated by team members.

[c] Rated by team leaders.

* $p < 0.05$

** $p < 0.01$.

https://doi.org/10.1371/journal.pone.0269196.t004

3.7 Descriptive statistics and correlation analysis

Means, standard deviations and correlations are shown in Table 4. Different effects of emotional labour were found; deep acting on taking charge (r = .468, p< .001), surface acting on taking charge (r = .007), deep acting on creative performance (r = .496, p = .001) and task performance (r = .405, p = .001), and surface acting on task performance (r = .178, p = .05) and creative performance (r = .154). In addition, analysis determined positive relationship between taking charge and creative performance (r = .425, p = .001) and task performance (r = .393, p< .001).

Meanwhile, control variables, age, and tenure were significantly related to one of our dependent variables, creative performance Age was significantly associated with other variables as well; deep acting and performance-based pay. Therefore, to evade a pointless decline in statistical power, we did not include control variables in regression analysis, which were not related to our dependent variables, as [94] recommended.

3.8 Test of mediation

Table 5 shows direct effect and path analysis outcomes for Hypotheses 1 to 3. In regards to Hypothesis 1a and Hypothesis 1b, as specified by significant unstandardized regression coefficient, findings state that deep acting affects taking charge positively (β = .517, t = 6.199, p< .01), but surface acting does not affect taking charge negatively (β = .005, t = .085, p = .932). Besides, Hypothesis 2 (a, b, c and d) indicates that deep acting is positively related to creative performance (H2a) (β = .812, t = 12.364, p< .01) and task performance (H2b) (β = .490, t = 6.666, p< .01), and surface acting is positively related to task performance (H2c) (β = .116, t = 2.116 $p < .05$). Whereas, surface acting is insignificantly related to creative performance (H2d) (β = .113, t = 1.824, p = .070). Therefore, Hypothesis 1 and Hypothesis 2 received partial support, because H1b and H2d did not meet expected results. At last, in support of Hypotheses 3a, 3b, and 3c, deep acting found to have an indirect effect on creative performance (3a) and task performance (3b), and effect of deep acting via taking charge on creative performance was stronger than the effect on task performance (3c). These indirect effects, as we assumed

Table 5. Regression results for simple mediation.

Regression results for the direct effect	***B***	***SE***	***t Value***	***Sig.***
Deep Acting—-→ Taking Charge	.517	.083	6.199	.000
Surface Acting—-→ Taking Charge	.005	.062	.085	.932
Deep Acting—-→ Creative Performance	.812	.066	12.364	.000
Deep Acting—-→ Task Performance	.490	.074	6.666	.000
Surface Acting—-→ Task Performance	.116	.055	2.116	.036
Surface Acting—-→ Creative Performance	.113	.062	1.824	.070
Regression results for simple mediation	***B***	***SE***	***t Value***	***Sig.***
Deep Acting—-→ Creative Performance	.812	.066	12.364	.000
Deep Acting—-→ Task Performance	.490	.074	6.666	.000
Deep Acting—-→ Taking Charge	.514	.083	6.175	.000
Taking Charge—-→ Creative performance	.371	.062	5.999	.000
Deep Acting—-→ Taking Charge—-→ Creative performance	.629	.067	9.349	.000
Taking Charge—-→ Task Performance	.303	.074	4.094	.000
Deep Acting—-→ Taking Charge—-→ Task Performance	.345	.081	4.281	.000

Bootstrap results for the indirect effect

	Effect	SE	LL 99% CI	UL 99% CI
Taking charge (Creative performance)	.191	.066	.079	.335
Taking charge (Task performance)	.156	.061	.054	.291

Notes: Sample size = 417 individuals from 186 teams; number of bootstraps resample = 10,000; LL = lower limit; UL = upper limit; CI = confidence interval

https://doi.org/10.1371/journal.pone.0269196.t005

(hypotheses 3a, 3b, and 3c), were positive for creative performance (.191) and task performance (.155). Bootstrap results demonstrated (Table 4) with bootstrapped 99% confidence interval around indirect effects are not containing zero for creative performance (.079, .345) and task performance (.054, .291). Thus, all hypotheses received full support except Hypotheses 1 and 2. As such, Hypothesis 1b and Hypothesis H2d were rejected for not meeting the expected assumptions.

3.9 Test of moderation

Table 6 shows outcomes for Hypotheses 4a to 4d. Results pointed out that the interaction term between taking charge and performance-based pay on creative performance (β = -.106, t = -3.810. $p = < .01$) and on task performance (β = -.074, t = -2.189. $p = < .05$) were significant. To entirely support Hypothesis 4a and Hypothesis 4b, we applied conventional practices for plotting simple slopes (Figs 2 and 3) at one standard deviation above and below the mean of performance-based pay measure. The positive relationship between taking charge and creative performance weakened under conditions of high performance-based pay. However, a positive relationship between taking charge and task performance increased under the condition of high performance-based pay. Hence, both hypotheses (H4a and H4b) received statistical support.

In support of Hypothesis 4c and Hypothesis 4d, we checked conditional indirect effects as demonstrated in Table 6. Conditional indirect effects of creative performance were; low (t = 7.34, p < .01), mean (t = 6.218, p < .01) and high (t = 3.187, p < .01), and of task performance were; low (t = 4.716, p < .01), mean (t = 4.149, p < .01) and high (t = 2.315, p < .05). All were significant under all three conditions. In sum, these significant indirect effects indicate that low performance-based pay strengthens the mediation effect of taking charge between deep acting and creative performance, which was consistent with Hypothesis 4c. In

Table 6. Regression results for the conditional indirect effect.

Predictor		***B***	***SE***	***t***	***p***
Taking Charge					
Constant		1.903	.669	2.845	.005
Deep acting		.514	.083	6.175	.000
Creative Performance					
Constant		-1.731	.763	-2.269	.025
Deep acting		.539	.069	7.853	.000
Taking charge		.887	.148	5.991	.000
Performance-based pay		.613	.151	4.052	.000
Taking charge **x** performance-based pay		-.106	.028	-3.810	.000
Task Performance					
Constant		.620	.928	.669	.505
Deep Acting		.239	.084	2.866	.005
Taking charge		.661	.180	3.670	.000
Performance-based pay		.533	.184	2.903	.004
Taking charge **x** performance-based pay		-.074	.034	-2.189	.030
**Moderator**	**Levels**	**Boot Indirect Effect**	**Boot SE**	**Boot *t***	**Boot *p***
Performance-based pay (Creative Perf.)	Low	.507	.069	7.354	.000
	Mean	.365	.587	6.218	.000
	High	.223	.701	3.187	.002
Performance-based pay (Task Perf.)	Low	.395	.084	4.716	.000
	Mean	.296	.071	4.149	.000
	High	.197	.085	2.315	.022

Notes: Sample size: 417 individuals from 186 teams; number of bootstraps resample = 10,000

https://doi.org/10.1371/journal.pone.0269196.t006

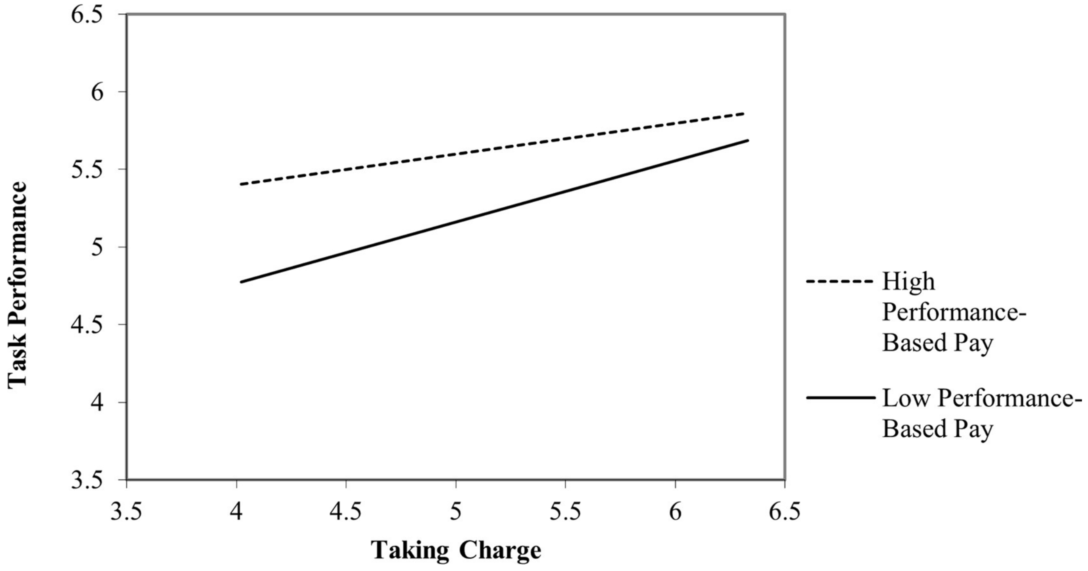

Fig 2. The moderating effect of performance-based pay on the relationship between taking charge and creative performance.

https://doi.org/10.1371/journal.pone.0269196.g002

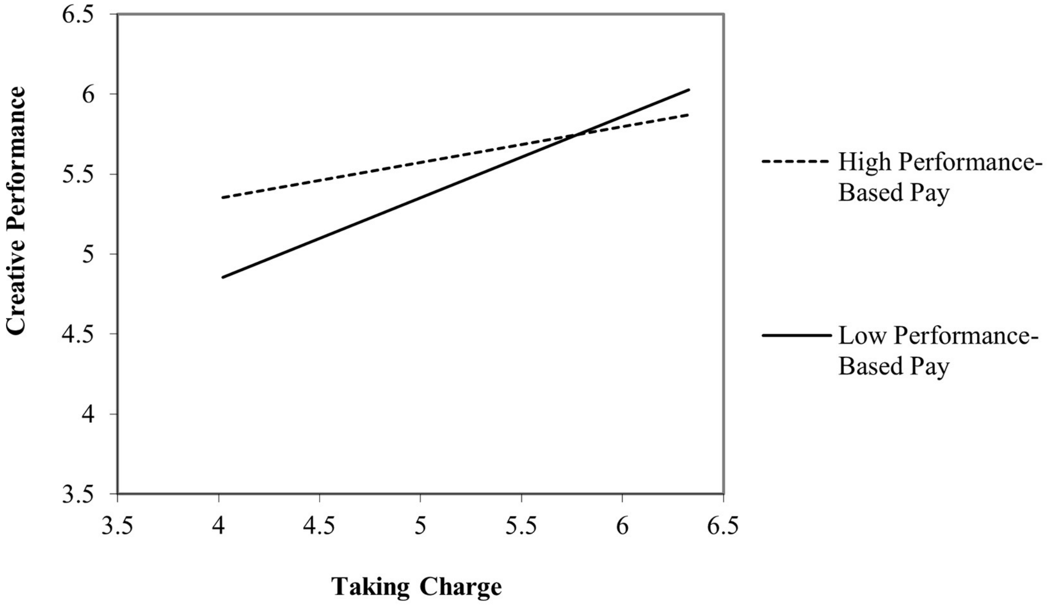

Fig 3. The moderating effect of performance-based pay on the relationship between taking charge and task performance.

https://doi.org/10.1371/journal.pone.0269196.g003

contrast, the mediation effect of taking charge between deep acting and task performance under high performance-based pay did not meet the expectation of Hypothesis 4d. Hence, H4c received support and H4d received rejection statistically.

4. Discussion, implications, and limitations

4.1 Discussion and theoretical implication

The importance of emotional labouring and performance of frontline service employees, who in their boundary-spanning positions significantly affect service-rendering organisations' efficiency by their direct communications with clients and customers, continues to increase. The key objective of current research has been to identify the behavioural mechanism between emotional labour and creative and task performance in service organisations (i.e., the banking sector). Drawing on self-determination theory, our study first examined how types of emotional labour differently promoted taking charge behaviour and performance (creative and task), by considering both types as motivation-based strategies (autonomous and controlled motivation). Later, we identified intervening factors in relationships between emotional labour strategies and creative and task performance. Finally, we investigated influencing factors to sustain the relationships on direct and indirect pathways.

We found that deep acting positively affected taking charge, while surface acting had no association with taking charge. Accordingly, H1a was supported and H1b was rejected, because it neither had a negative relationship nor a positive one. We also found deep acting positively affected creative performance, while surface acting was not associated–positively or negatively–with creative performance. However, both emotional labour strategies demonstrated a positive relationship with task performance. As a result, hypotheses H2a, H2b, and H2c were supported, and H2d was rejected. Later, we examined the effect of deep acting on creative performance and task performance by bringing in the mediating role of taking charge. Our study confirmed that emotional labour (deep acting) not only directly manipulated creative performance and task performance but also indirectly assisted creative performance (H3a)

and task performance (H3b) by improving taking charge behaviours. Furthermore, it also clarified that the effect of taking charge on creative performance was stronger than the effect on task performance, which supported H3c. As a result, our study offers several significant contributions.

For example, our study attempts to disclose how taking charge, fueled by emotional labour (deep acting), directs employees' voluntary efforts towards creative performance and task performance. Consistent with past studies, our findings show that a deep acting strategy significantly affected creative performance and task performance. Scholars have found that a deep acting strategy can directly enhance creativity and job performance by encouraging employees to control their emotional exhaustion and channel their attention towards commitment and work engagement [15, 24]. However, it is essential to expand on these surprising outcomes, specifically in the banking sector and in a collectivist society like Pakistan [95]. In the past few years, several studies have been conducted e.g., [96–99] to identify emotional labour and individual responses in the hospital industry and the educational sector in Pakistan. Therefore, our outcomes regarding the different effects of emotional labour on taking charge, the direct association between emotional labour and job performance, and the mediation of taking charge demonstrated an exceptional contribution to the literatures of emotional labour, proactive behaviours, and creativity theory in the context of the banking sector and a collectivist society. The outcomes demonstrated that deep acting was an antecedent of taking charge behaviour, and it manipulated job performance directly as well as indirectly. This confirms the partial mediation of taking charge.

But, past studies have shown a negative relationship between surface acting and task performance [22, 27, 100]. Our findings, however, contradicted these findings and demonstrated that surface acting had a positive relationship with task performance from the perspective of self-determination theory. This outcome is consistent with hypothesis H2c and adds a new direction for researchers. The reason for this contradictory finding might be that when individuals feel controlled, they experience pressure to think, behave, or feel in specific ways [51] and perform as per given instructions to avoid punishment. In other words, the individuals abide by demands in the hope of attaining an imagined endorsement and thus feel sheltered [101, 102]. Likewise, according to Judge et al. [66] and Pugh, Groth, and Henning-Thurau [67], surface acting may not be detrimental when the surface actor is socially skilled at appearing authentic or systematically managed. Therefore, this finding supports the claims of Judge et al. [66] and Pugh, Groth, and Henning-Thurau [67] by demonstrating a significant link between surface acting and task performance.

In addition, our research also interprets the outcomes of taking charge from the perception of performance-based pay. In other words, our findings clarify how performance-based pay promotes or restrains taking charge on job performance. In particular, our study demonstrated that performance-based pay directly and indirectly moderated the relationship. For instance, the direct relationship between taking charge and creative performance and the direct link between taking charge and task performance was statistically significant. These results supported hypotheses H4a and H4b. Past studies focused mostly on pay, emotional competence, and organisational tenure as the moderator through which they controlled taking charge e.g., [38, 47, 48]. However, our results showed that performance-based pay acted as a moderating mechanism influencing taking charge behaviour with creative and task performance. Hence, our findings suggest that one key mechanism by which taking charge can affect job performance is by introducing a compensation system based on motivational level. Noticeably, these findings regarding financial incentives align with past studies that demonstrated that high pay can increase taking charge behaviour [38] and task performance [43]. But when it comes to creativity, transactional incentives typically do not encourage creativity influenced by autonomous motivation [39, 43, 46, 103].

Moreover, this study also confirmed that the indirect relationship between deep acting and creative and task performance through taking charge was dependent upon the level of performance-based pay. As per the assumptions in hypothesis H4c, high performance-based pay lowered the indirect relationship between deep acting and creative performance via taking charge. Hypothesis H4c was supported as the boot indirect effect was .223 at high-level. We then hypothesised (H4d) that high performance-based pay strengthened the indirect relationship between deep acting and task performance via taking charge. The indirect relationship between deep acting and task performance through taking charge did not receive support. As per results, the boot indirect effect was .197 at high-level, which is low in comparison to a low-level boot indirect effect .395. Thus, hypothesis H4d was rejected. Our study confirmed that emotional labour (deep acting) had an indirect positive link with creative and task performance via taking charge at all levels of performance-based pay. However, low performance-based pay had a strong effect. Therefore, the outcomes contribute significant empirical evidence to support the claim that, under the right conditions (e.g., low performance-based pay), a deep acting strategy can be a crucial activator of voluntary or extra-role behaviour, and, in the process, it can promote taking charge behaviour that leads to creative and task performance; see, [19, 20, 25, 41, 104].

4.2 Managerial implications

Our findings suggest that emotional labour (deep acting) is positively related to taking charge and creative and task performance. Employees with deep acting expend more effort, take charge voluntarily, and display creativity in their work. Thus, organisations should take an employee's deep acting into account when determining whether he/she is inherently motivated towards the job, and pay more attention during the recruitment process and talent hunt programmes. It might increase the chances of accomplishing an organisation's goals for innovation and lay a foundation for firms to acquire higher taking charge at the commencement of talent introduction. Also, when individuals are chosen for qualities that are associated with a job's display rules (e.g., high self-control, job identity, and positive affectivity), emotional labour is most likely done with deep acting.

According to Judge et al. [66] and Pugh, Groth, and Henning-Thurau [67], surface acting is not always detrimental. For example, a surface actor may be socially skilled at appearing authentic and managed systematically. Thus, organisations can train them, counsel them, or manage them in such a way that favors them in the long run such that deep acting could be learnt [12]. In doing so, a surface actor can turn out to be deep actor. Consequently, organisations should arrange training sessions to help their staff master their skills and approaches towards deep acting strategy.

Additionally, to let employees be creative, organisations should introduce creativity-contingent rewards to encourage individuals towards creative performance. According to Byron [103], creativity-contingent rewards have a tendency to improve creative performance rather than performance-contingent rewards. Further, employees should be given task-focused performance feedback, and they should be less controlled while working. This will provide them freedom of choice, and they will be more motivated and dedicated towards organisational goals.

4.3 Limitations and future directions

While this research contributes to the literature, it has some limitations as well. First, the use of self-reported survey data may have introduced biases. Although, our research was cross-sectional at different time points from two different sources to enhance the validity of our

outcomes, we admit that strong causal inferences might be problematic [105]. This issue is specifically applicable to taking charge, which is based on self-reported assessments from one source only. In this case, we advise future researchers to consider multiple sources of data to tackle issues of causality and self-reporting biases. But favoritism could be a reason for different ratings for different individuals by the team leader. Therefore, it is essential to consider multiple sources for data collection. Additionally, our outcomes focused exclusively on front-line employees in local banks in Pakistan, which may not be generalizable to other service organisations or cultural contexts. Thus, scholars may consider these shortcomings, and attempt to replicate our findings by studying other service organisations and performing cross-cultural research.

Future studies should consider examining the relationship conflict, social or formal status, and emotional intelligence to influence our model. We also advise future scholars to consider a country's economy to identify the motivation level of emotional labour–specifically, is it autonomous motivation or controlled motivation when emotional labour fosters a creative and proactive approach? Answering this question might provide insight about emotional labouring in other developing countries with unstable economic conditions. In a similar vein, past studies have shown a positive relationship between surface acting and emotional exhaustion and stress [27, 37, 106, 107] the positive effect of surface acting on task performance–specifically, how long will a surface acting be associated with task performance positively?

Author Contributions

Conceptualization: Nilesh Kumar, Zhiqiang Liu, Carol Flinchbaugh, Md. Yahin Hossain, Md. Nahin Hossain.

Data curation: Nilesh Kumar.

Formal analysis: Nilesh Kumar, Md. Yahin Hossain.

Funding acquisition: Nilesh Kumar.

Investigation: Nilesh Kumar.

Methodology: Nilesh Kumar, Zhiqiang Liu, Carol Flinchbaugh.

Project administration: Zhiqiang Liu.

Software: Nilesh Kumar, Md. Yahin Hossain, Md. Nahin Hossain.

Supervision: Zhiqiang Liu, Carol Flinchbaugh.

Validation: Nilesh Kumar, Md. Nahin Hossain.

Visualization: Nilesh Kumar, Md. Yahin Hossain, Md. Nahin Hossain.

Writing – original draft: Nilesh Kumar.

Writing – review & editing: Nilesh Kumar, Zhiqiang Liu, Carol Flinchbaugh.

References

1. Agnihotri R., Rapp A. A., 'Mick' Andzulis J., and Gabler C. B., "Examining the drivers and performance implications of boundary spanner creativity," *J. Serv. Res.*, vol. 17, no. 2, pp. 164–181, 2014.
2. Fernandes T. and Pinto T., "Relationship quality determinants and outcomes in retail banking services: The role of customer experience," *J. Retail. Consum. Serv.*, vol. 50, pp. 30–41, 2019.
3. Berry L. L., Shankar V., Parish J. T., Cadwallader S., and Dotzel T., "Creating new markets through service innovation," *MIT Sloan Manag. Rev.*, vol. 47, no. 2, p. 56, 2006.

4. Cadwallader S., Jarvis C. B., Bitner M. J., and Ostrom A. L., "Frontline employee motivation to participate in service innovation implementation," *J. Acad. Mark. Sci.*, vol. 38, no. 2, pp. 219–239, 2010.
5. Coelho F., Augusto M., and Lages L. F., "Contextual factors and the creativity of frontline employees: The mediating effects of role stress and intrinsic motivation," *J. Retail.*, vol. 87, no. 1, pp. 31–45, 2011.
6. Shalley C. E., "Creating roles: What managers can do to establish expectations for creative performance," *Handb. Organ. Creat.*, pp. 147–164, 2008.
7. Humphrey R. H., Ashforth B. E., and Diefendorff J. M., "The bright side of emotional labor," *J. Organ. Behav.*, vol. 36, no. 6, pp. 749–769, 2015, https://doi.org/10.1002/job
8. Ashforth B. E. and Humphrey R. H., "Emotional Labor in Service Roles: The Influence of Identity," *Acad. Manag. Rev.*, vol. 18, no. 1, pp. 88–115, 1993, https://doi.org/10.2307/258824
9. Grandey A. A., "Emotional regulation in the workplace: A new way to conceptualize emotional labor.," *J. Occup. Health Psychol.*, vol. 5, no. 1, p. 95, 2000.
10. Grandey A. A., "When 'The Show Must Go On': Surface Acting and Deep Acting as Determinants of Emotional Exhaustion and Peer-Rated Service Delivery," *Acad. Manag. J.*, vol. 46, no. 1, pp. 86–96, Feb. 2003, https://doi.org/10.5465/30040678
11. Hochschild Arlie R., "The managed heart: Commercialization of human feeling." Berkeley, University of California Press, 1983.
12. Morris J. A. and Feldman D. C., "The dimensions, antecedents, and consequences of emotional labor," *Acad. Manag. Rev.*, vol. 21, no. 4, pp. 986–1010, 1996.
13. Geng Z., Liu C., Liu X., and Feng J., "The effects of emotional labor on frontline employee creativity," *Int. J. Contemp. Hosp. Manag.*, vol. 26, no. 7, pp. 1046–1064, 2014, https://doi.org/10.1108/IJCHM-12-2012-0244
14. Liu C., Liu X., and Geng Z., "Emotional labor strategies and service performance: The mediating role of employee creativity," *J. Appl. Bus. Res.*, vol. 29, no. 5, pp. 1583–1596, 2013, https://doi.org/10.19030/jabr.v29i5.8076
15. Shin I., Hur W.-M., and Oh H., "Essential precursors and effects of employee creativity in a service context," *Career Dev. Int.*, 2015, https://doi.org/10.1108/CDI-10-2014-0137
16. Karim J. and Weisz R., "Emotional Intelligence as a Moderator of Affectivity/Emotional Labor and Emotional Labor/Psychological Distress Relationships," *Psychol. Stud.*, vol. 56, no. 4, p. 348, 2011, https://doi.org/10.1007/s12646-011-0107-9
17. Cropanzano R., Weiss H. M., and Elias S. M., *the Impact of Display Rules and Emotional Labor on Psychological Well-Being At Work*, vol. 3. 2003.
18. Grandey A. A., Fisk G. M., Mattila A. S., Jansen K. J., and Sideman L. A., "Is 'service with a smile' enough? Authenticity of positive displays during service encounters," *Organ. Behav. Hum. Decis. Process.*, vol. 96, no. 1, pp. 38–55, 2005, https://doi.org/10.1016/j.obhdp.2004.08.002
19. Hülsheger U. R., Schewe A. F., Hu U. R., and Schewe A. F., "On the costs and benefits of emotional labor: A meta-analysis of three decades of research.," *J. Occup. Health Psychol.*, vol. 16, no. 3, pp. 361–389, 2011, https://doi.org/10.1037/a0022876 PMID: 21728441
20. Ramachandran Y., Jordan P. J., Troth A. C., and Lawrence S. A., "Emotional Intelligence, Emotional Labour and Organisational Citizenship Behaviour in service environments," *Int. J. Work Organ. Emot.*, vol. 4, no. 2, p. 136, 2012, https://doi.org/10.1504/ijwoe.2011.044594
21. M. Torland, "Emotional labour and the job satisfaction of adventure tour leaders in Australia Emotional Labour and the Job Satisfaction of Adventure Tour Leaders in Australia," 2013.
22. Bağcı Z., Bayrak Kök S., and Mohan Bursalı Y., "The relationship between emotional labor and task/contextual/innovative job performance: A study with private banking employees in Denizli," *Eur. J. Res. Educ.*, vol. 2, no. 2, pp. 221–221, 2014, https://doi.org/10.15527/ejre.201426267
23. Hameed F., Anwar M. M., and Ahmed J., "Emotional Labor and Creativity: Testing a Moderated-Mediated Model in Banking Sector.," *Dialogue (Pakistan)*, vol. 14, no. 3, 2019.
24. Jaewon Y., Jicheol J., and Authors F., "The effects of emotional labor on work engagement and boundary spanner creativity," *Asia Pacific J. Innov. Entrep.*, vol. 11, no. 2, pp. 214–232, Jan. 2017, https://doi.org/10.1108/APJIE-08-2017-028
25. Grandey A. A. and Sayre G. M., "Emotional Labor: Regulating Emotions for a Wage," 2019, https://doi.org/10.1177/0963721418812771
26. Zhou J. and George J. M., "Awakening employee creativity: The role of leader emotional intelligence," *Leadersh. Q.*, vol. 14, no. 4, pp. 545–568, 2003, https://doi.org/10.1016/S1048-9843(03)00051-1.
27. Tracy S., Chris Z., and Huo Y., "Antecedents and outcomes of emotional labour in hospitality and tourism: A meta-analysis," *Tour. Manag.*, vol. 79, no. August 2019, p. 104099, 2020, https://doi.org/10.1016/j.tourman.2020.104099

28. Deci E. L. and Ryan R. M., *Intrinsic Motivation and Self-Determination in Human Behavior*. 1985.

29. Cangiano F., Parker S. K., and Ouyang K., "Too proactive to switch off: When taking charge drains resources and impairs detachment.," *J. Occup. Health Psychol.*, no. October, 2020, https://doi.org/10.1037/ocp0000265 PMID: 33104372

30. Morrison E. W. and Phelps C. C., "Taking charge at work: Extrarole efforts to initiate workplace change," *Acad. Manag. J.*, vol. 42, no. 4, pp. 403–419, 1999, https://doi.org/10.2307/257011

31. Kumar N., Liu Z., and Jin Y., "Evaluation of Employee Empowerment on Taking Charge Behaviour: An Application of Perceived Organizational Support as a Moderator," *Psychol. Res. Behav. Manag.*, vol. 15, no. April, pp. 1055–1066, 2022. https://doi.org/10.2147/PRBM.S355326 PMID: 35517430

32. Hogg M. A. and Cooper J., *The SAGE handbook of social psychology*: *Concise student edition*. Sage, 2007.

33. Katz D., "The functional approach to the study of attitudes," *Public Opin. Q.*, vol. 24, no. 2, pp. 163–204, 1960.

34. Kumar N., Hossain M. Y., Jin Y., Safeer A. A., and Chen T., "Impact of Performance Lower Than Expectations on Work Behaviors: The Moderating Effect of Status Mutability and Mediating Role of Regulatory Focus," *Psychol. Res. Behav. Manag.*, vol. Volume 14, no. December, pp. 2257–2270, Dec. 2021, https://doi.org/10.2147/PRBM.S342562 PMID: 35002343

35. Cossette M., "Emotional labor through the lens of self-determination theory," *Oxford Handb. Work Engag. Motiv. Self-Determination Theory*, *Oxford Univ. Press.* New York, NY, pp. 259–275, 2014.

36. Gagne M., "Autonomy support and need satisfaction in the motivation and well-being of gymnasts," *J. Appl. Sport Psychol.*, vol. 15, no. 4, pp. 372–390, 2003.

37. Hur W. M., Shin Y., and Moon T. W., "Linking Motivation, Emotional Labor, and Service Performance From a Self-Determination Perspective," *J. Serv. Res.*, 2020, https://doi.org/10.1177/1094670520975204

38. El Baroudi S. et al., "Ambition at work and career satisfaction The mediating role of taking charge behavior," 2017, https://doi.org/10.1108/CDI-07-2016-0124

39. Fang M. and Gerhart B., "Does pay for performance diminish intrinsic interest?," *Int. J. Hum. Resour. Manag.*, vol. 23, no. 6, pp. 1176–1196, Mar. 2012, https://doi.org/10.1080/09585192.2011.561227

40. Ryan R. M. and Deci E. L., *Self-determination theory*: *Basic psychological needs in motivation*, *development*, *and wellness*. New York, NY, US: Guilford Press, 2017.

41. Kumar N., Zhiqiang L., and Hossain M. Y., "Examining the relationship between self-actualization and job performance via taking charge," *Int. J. Res. Bus. Soc. Sci. (2147–4478)*, vol. 9, no. 5 SE-Managerial Issues in Organizational Development and Human Resources Management, pp. 43–49, Sep. 2020, https://doi.org/10.20525/ijrbs.v9i5.858

42. Burleson W., "Developing creativity, motivation, and self-actualization with learning systems," *Int. J. Hum. Comput. Stud.*, vol. 63, no. 4–5 SPEC. ISS., pp. 436–451, 2005, https://doi.org/10.1016/j.ijhcs.2005.04.007

43. Fischer C., Malycha C. P., and Schafmann E., "The Influence of Intrinsic Motivation and Synergistic Extrinsic Motivators on Creativity and Innovation," *Front. Psychol.*, vol. 10, no. February, p. 137, 2019, https://doi.org/10.3389/fpsyg.2019.00137 PMID: 30778313

44. Rahman M. A., Butt A. N., and Choi J. N., "Rewards and employee creative performance: Moderating effects of creative self-efficacy, reward importance, and locus of control," *J. Organ. Behav.*, vol. 36, no. 1, pp. 59–74, Jan. 2015, https://doi.org/10.1002/job.1943

45. Byron K. and Khazanchi S., "Rewards and Creative Performance: A Meta-Analytic Test of Theoretically Derived Hypotheses," *Psychol. Bull.*, vol. 138, no. March 2012, pp. 809–830, Mar. 2012, https://doi.org/10.1037/a0027652 PMID: 22409506

46. Cerasoli C. P., Nicklin J. M., Ford M. T., Cerasoli C. P., Nicklin J. M., and Ford M. T., "Intrinsic motivation and extrinsic incentives jointly predict performance: A 40-year meta-analysis.," *Psychol. Bull.*, vol. 140, no. 4, pp. 980–1008, 2014, https://doi.org/10.1037/a0035661 PMID: 24491020

47. Kim T.-Y., Liu Z., and Diefendorff J. M., "Leader–member exchange and job performance: The effects of taking charge and organizational tenure," *J. Organ. Behav.*, vol. 36, no. 2, pp. 216–231, Feb. 2015, https://doi.org/10.1002/job.1971

48. Kim T.-Y. and Liu Z., "Taking charge and employee outcomes: the moderating effect of emotional competence," *Int. J. Hum. Resour. Manag.*, vol. 28, no. 5, pp. 775–793, Mar. 2017, https://doi.org/10.1080/09585192.2015.1109537

49. Deci E. L. and Gagné M., "Self-determination theory and work motivation," *J. Organ. Behav.*, vol. 26, no. 4, pp. 331–362, 2005, https://doi.org/10.1002/job.322

50. Deci E. L. and Ryan R. M., "The 'What' and 'Why' of Goal Pursuits: Human Needs and the Self-Determination of Behavior," *Psychol. Inq.*, vol. 11, no. 4, pp. 227–268, Oct. 2000, https://doi.org/10.1207/S15327965PLI1104_01

51. Deci E. L. and Ryan R. M., "Self-determination theory: A macrotheory of human motivation, development, and health.," *Can. Psychol. Can.*, vol. 49, no. 3, pp. 182–185, 2008, https://doi.org/10.1037/a0012801

52. Ryan R. M. and Connell J. P., "Perceived locus of causality and internalization: examining reasons for acting in two domains.," *J. Pers. Soc. Psychol.*, vol. 57, no. 5, p. 749, 1989. https://doi.org/10.1037//0022-3514.57.5.749 PMID: 2810024

53. Rubin R. S., Tardino V. M. S., Daus C. S., and Munz D. C., "A reconceptualization of the emotional labor construct: On the development of an integrated theory of perceived emotional dissonance and emotional labor.," 2005.

54. Brotheridge C. M. and Lee R. T., "Testing a conservation of resources model of the dynamics of emotional labor.," *Journal of Occupational Health Psychology*, vol. 7, no. 1. Educational Publishing Foundation, Brotheridge, Céeste M.: U Regina, Faculty of Administration, Room 456, Education Building, Regina, SK, Canada, S4S 0A2, celeste.brotheridge@uregina.ca, pp. 57–67, 2002, https://doi.org/10.1037/1076-8998.7.1.57

55. Chi N.-W. and Grandey A. A., "Emotional Labor Predicts Service Performance Depending on Activation and Inhibition Regulatory Fit," *J. Manage.*, vol. 45, no. 2, pp. 673–700, Oct. 2016, https://doi.org/10.1177/0149206316672530

56. Fuller J. B., Barnett T., Hester K., Relyea C., and Frey L., "An exploratory examination of voice behavior from an impression management perspective," *J. Manag. Issues*, 2007, https://doi.org/10.2307/40601197

57. Fuller B. and Marler L. E., "Change driven by nature: A meta-analytic review of the proactive personality literature," *J. Vocat. Behav.*, vol. 75, no. 3, pp. 329–345, 2009, https://doi.org/10.1016/j.jvb.2009.05.008

58. Wu C. H., Parker S. K., Wu L. Z., and Lee C., "When and why people engage in different forms of proactive behavior: Interactive effects of self-construals and work characteristics," *Acad. Manag. J.*, vol. 61, no. 1, pp. 293–323, 2018, https://doi.org/10.5465/amj.2013.1064

59. Grant A. M. and Ashford S. J., "The dynamics of proactivity at work," *Res. Organ. Behav.*, vol. 28, pp. 3–34, 2008, https://doi.org/10.1016/j.riob.2008.04.002

60. Coté S., "A social interaction model of the effects of emotion regulation on work strain," *Acad. Manag. Rev.*, vol. 30, no. 3, pp. 509–530, 2005.

61. Koestner R., Bernieri F., and Zuckerman M., "Self-regulation and consistency between attitudes, traits, and behaviors," *Personal. Soc. Psychol. Bull.*, vol. 18, no. 1, pp. 52–59, 1992.

62. Love M. S. and Dustin S. L., "An investigation of coworker relationships and psychological collectivism on employee propensity to take charge," *Int. J. Hum. Resour. Manag.*, vol. 25, no. 9, pp. 1208–1226, May 2014, https://doi.org/10.1080/09585192.2013.826712

63. Oldham G. R. and Cummings A., "Employee Creativity: Personal and Contextual Factors at Work," *Acad. Manag. J.*, vol. 39, no. 3, pp. 607–634, Jun. 1996, https://doi.org/10.5465/256657

64. Meyer J. P., Stanley D. J., Herscovitch L., and Topolnytsky L., "Affective, continuance, and normative commitment to the organization: A meta-analysis of antecedents, correlates, and consequences," *J. Vocat. Behav.*, vol. 61, no. 1, pp. 20–52, 2002.

65. Harari M. B., Reaves A. C., and Viswesvaran C., "Creative and innovative performance: a meta-analysis of relationships with task, citizenship, and counterproductive job performance dimensions," *Eur. J. Work Organ. Psychol.*, vol. 25, no. 4, pp. 495–511, 2016, https://doi.org/10.1080/1359432X.2015.1134491

66. Judge T. A., Woolf E. F., and Hurst C., "Is emotional labor more difficult for some than for others? A multilevel, experience-sampling study," *Pers. Psychol.*, vol. 62, no. 1, pp. 57–88, 2009.

67. Pugh S. D., Groth M., and Hennig-Thurau T., "Willing and able to fake emotions: a closer examination of the link between emotional dissonance and employee well-being.," *J. Appl. Psychol.*, vol. 96, no. 2, p. 377, 2011. https://doi.org/10.1037/a0021395 PMID: 21058805

68. Milkovich G. and Milkovich C., "Strengthening the pay-performance relationship: The research," *Compens. Benefits Rev.*, vol. 24, no. 6, pp. 53–62, 1992.

69. Appelbaum L. G., Schroeder J. E., Cain M. S., and Mitroff S. R., "Improved visual cognition through stroboscopic training," *Front. Psychol.*, vol. 2, p. 276, 2011. https://doi.org/10.3389/fpsyg.2011.00276 PMID: 22059078

70. Lemieux T., MacLeod W. B., and Parent D., "Performance pay and wage inequality," *Q. J. Econ.*, vol. 124, no. 1, pp. 1–49, 2009.

71. Khan K. U., Farooq S. U., and Ullah M. I., "The relationship between rewards and employee motivation in commercial banks of Pakistan," *Res. J. Int. Stud.*, vol. 14, pp. 37–52, 2010.

72. Chng D. H. M. and Wang J. C. Y., "An experimental study of the interaction effects of incentive compensation, career ambition, and task attention on Chinese managers' strategic risk behaviors," *J. Organ. Behav.*, vol. 37, no. 5, pp. 719–737, 2016.

73. Vadera A. K., Pratt M. G., and Mishra P., "Constructive Deviance in Organizations: Integrating and Moving Forward," *J. Manage.*, 2013, https://doi.org/10.1177/0149206313475816

74. Cai Z., Huo Y., Lan J., Chen Z., and Lam W., "When Do Frontline Hospitality Employees Take Charge? Prosocial Motivation, Taking Charge, and Job Performance: The Moderating Role of Job Autonomy," *Cornell Hosp. Q.*, vol. 60, no. 3, pp. 237–248, 2019, https://doi.org/10.1177/1938965518797081

75. Deckop J. R., Merriman K. K., and Blau G., "Impact of variable risk preferences on the effectiveness of control by pay," *J. Occup. Organ. Psychol.*, vol. 77, no. 1, pp. 63–80, 2004.

76. Azoulay P., Graff Zivin J. S., and Manso G., "Incentives and creativity: evidence from the academic life sciences," *RAND J. Econ.*, vol. 42, no. 3, pp. 527–554, 2011.

77. Ederer F. and Manso G., "Is pay for performance detrimental to innovation?," *Manage. Sci.*, vol. 59, no. 7, pp. 1496–1513, 2013.

78. Zhang Y., Long L., and Zhang J., "Pay for performance and employee creativity," *Manag. Decis.*, vol. 53, no. 7, pp. 1378–1397, 2015, https://doi.org/10.1108/md-11-2013-0596

79. Deci E. L. and Ryan R. M., "The support of autonomy and the control of behavior.," *J. Pers. Soc. Psychol.*, vol. 53, no. 6, p. 1024, 1987. https://doi.org/10.1037//0022-3514.53.6.1024 PMID: 3320334

80. Eisenberger R. and Aselage J., "Incremental effects of reward on experienced performance pressure: Positive outcomes for intrinsic interest and creativity," *J. Organ. Behav. Int. J. Ind. Occup. Organ. Psychol. Behav.*, vol. 30, no. 1, pp. 95–117, 2009.

81. Shirom A., Westman M., and Melamed S., "The effects of pay systems on blue-collar employees' emotional distress: The mediating effects of objective and subjective work monotony," *Hum. Relations*, vol. 52, no. 8, pp. 1077–1097, 1999.

82. Amabile T. M., "Creativity in context (Boulder, CO, Westview Press)," 1996.

83. Kohn A., "Punished by Rewards: The Trouble with Gold Stars," 1999.

84. McGraw K. O., "The detrimental effects of reward on performance: A literature review and a prediction model," *hidden costs Reward New Perspect. Psychol. Hum. Motiv.*, pp. 33–60, 1978.

85. Preacher K. J. and Hayes A. F., "SPSS and SAS procedures for estimating indirect effects in simple mediation models," *Behav. Res. methods, instruments, Comput.*, vol. 36, no. 4, pp. 717–731, 2004. https://doi.org/10.3758/bf03206553 PMID: 15641418

86. Preacher K. J., Rucker D. D., and Hayes A. F., "Addressing moderated mediation hypotheses: Theory, methods, and prescriptions," *Multivariate Behav. Res.*, vol. 42, no. 1, pp. 185–227, 2007. https://doi.org/10.1080/00273170701341316 PMID: 26821081

87. Hayes A. F., *Introduction to mediation, moderation, and conditional process analysis: A regression-based approach.* Guilford publications, 2017.

88. Grant A. M., Gino F., and Hofmann D. A., "Reversing the extraverted leadership advantage: The role of employee proactivity," *Acad. Manag. J.*, vol. 54, no. 3, pp. 528–550, 2011.

89. van. Dyne L., Cummings L. L., and McLean Parks J., "Extra-role behaviors: in pursuit of construct and definitional clarity (a bridge over muddied waters)," Res. Organ. Behav. an Annu. Ser. Anal. essays Crit. Rev., vol. 17, p. 215, 1995.

90. Deckop J. R., Mangel R., and Cirka C. C., "Research Notes. Getting More Than You Pay For: Organizational Citizenship Behavior and Pay-For-Performance Plans," *Acad. Manag. J.*, vol. 42, no. 4, pp. 420–428, Aug. 1999, https://doi.org/10.5465/257012

91. Janssen O. and Van Yperen N. W., "Employees' Goal Orientations, the Quality of Leader-Member Exchange, and the Outcomes of Job Performance and Job Satisfaction," *Acad. Manag. J.*, vol. 47, no. 3, pp. 368–384, Jun. 2004, https://doi.org/10.5465/20159587

92. Williams L. J. and Anderson S. E., "Job satisfaction and organizational commitment as predictors of organizational citizenship and in-role behaviors," *J. Manage.*, vol. 17, no. 3, pp. 601–617, 1991.

93. HairJr J. F., Matthews L. M., Matthews R. L., and Sarstedt M., "PLS-SEM or CB-SEM: updated guidelines on which method to use," *Int. J. Multivar. Data Anal.*, vol. 1, no. 2, pp. 107–123, 2017.

94. Becker T. E., "Potential problems in the statistical control of variables in organizational research: A qualitative analysis with recommendations," *Organ. Res. Methods*, vol. 8, no. 3, pp. 274–289, 2005.

95. Hofstede G., "Motivation, leadership, and organization: Do American theories apply abroad?," *Organ. Dyn.*, vol. 9, no. 1, pp. 42–63, 1980, https://doi.org/10.1016/0090-2616(80)90013-3.

96. Asrar-ul-Haq M., Anwar S., and Hassan M., "Impact of emotional intelligence on teacher's performance in higher education institutions of Pakistan," *Futur. Bus. J.*, vol. 3, no. 2, pp. 87–97, 2017.

97. Hameed F., "Impact of Personality on Adaptive Performance with Mediating Role of Emotional Labor. Testing a Mediated Moderated Model among Front-line Female Hospitality Industry Employees in Pakistan." CAPITAL UNIVERSITY OF SCIENCE & TECHNOLOGY ISLAMABAD, 2016.

98. Khalil A., Khan M. M., Raza M. A., and Mujtaba B. G., "Personality Traits, Burnout, and Emotional Labor Correlation among Teachers in Pakistan," *J. Serv. Sci. Manag.*, vol. 10, no. 6, pp. 482–496, 2017.

99. Pervaiz S., Ali A., and Asif M., "Emotional intelligence, emotional labor strategies and satisfaction of secondary teachers in Pakistan," *Int. J. Educ. Manag.*, 2019.

100. Hülsheger U. R., Lang J. W. B., Maier G. W., Hu U. R., and Lang J. W. B., "Emotional labor, strain, and performance: Testing reciprocal relationships in a longitudinal panel study," *J. Occup. Health Psychol.*, vol. 15, no. 4, p. 505–521, Oct. 2010, https://doi.org/10.1037/a0021003 PMID: 21058862

101. Freud S., "The economic problem of masochism," in *The Standard Edition of the Complete Psychological Works of Sigmund Freud, Volume XIX (1923–1925): The Ego and the Id and Other Works*, 1961, pp. 155–170.

102. Meissner W. W., "Internalization in Psychoanalysis New York: Int." Univ. Press, 1981.

103. K. Byron, "Rewards and Creative Performance: A Meta-Analytic Test of Theoretically Rewards and Creative Performance: A Meta-Analytic Test of Theoretically Derived Hypotheses," no. March 2012, 2015, https://doi.org/10.1037/a0027652

104. Li X., "The Preliminary Literature Review of Proactive Behavior," *Am. J. Ind. Bus. Manag.*, vol. 10, no. 05, pp. 915–919, 2020, https://doi.org/10.4236/ajibm.2020.105061

105. Chen G. et al., "The Power of Momentum: A New Model of Dynamic Relationships between Job Satisfaction Change and Turnover Intentions," *Acad. Manag. J.*, vol. 54, no. 1, pp. 159–181, Feb. 2011, https://doi.org/10.5465/amj.2011.59215089

106. Jeung D. Y., Kim C., and Chang S. J., "Emotional labor and burnout: A review of the literature," *Yonsei Med. J.*, vol. 59, no. 2, pp. 187–193, 2018, https://doi.org/10.3349/ymj.2018.59.2.187 PMID: 29436185

107. H. Kong and J. Jeon, "Daily Emotional Labor, Negative Affect State, and Emotional Exhaustion: Cross-Level Moderators of Affective Commitment," 2018, https://doi.org/10.3390/su10061967
